# Histidine Prevents Cu-Induced Oxidative Stress and the Associated Decreases in mRNA from Encoding Tight Junction Proteins in the Intestine of Grass Carp (*Ctenopharyngodon idella*)

**DOI:** 10.1371/journal.pone.0157001

**Published:** 2016-06-09

**Authors:** Wei-Dan Jiang, Biao Qu, Lin Feng, Jun Jiang, Sheng-Yao Kuang, Pei Wu, Ling Tang, Wu-Neng Tang, Yong-An Zhang, Xiao-Qiu Zhou, Yang Liu

**Affiliations:** 1 Animal Nutrition Institute, Sichuan Agricultural University, Chengdu, 611130, Sichuan, China; 2 Fish Nutrition and Safety Production University Key Laboratory of Sichuan Province, Sichuan Agricultural University, Chengdu, 611130, Sichuan, China; 3 Key Laboratory for Animal Disease-Resistance Nutrition of China Ministry of Education, Sichuan Agricultural University, Chengdu, 611130, Sichuan, China; 4 Animal Nutrition Institute, Sichuan Academy of Animal Science, 610066, Chengdu, China; 5 Institute of Hydrobiology, Chinese Academy of Sciences, Wuhan, 430072, China; CINVESTAV-IPN, MEXICO

## Abstract

Copper (Cu) is a common heavy metal pollutant in aquatic environments that originates from natural as well as anthropogenic sources. The present study investigated whether Cu causes oxidative damage and induces changes in the expression of genes that encode tight junction (TJ) proteins, cytokines and antioxidant-related genes in the intestine of the grass carp (*Ctenopharyngodon idella*). We demonstrated that Cu decreases the survival rate of fish and increases oxidative damage as measured by increases in malondialdehyde and protein carbonyl contents. Cu exposure significantly decreased the expression of genes that encode the tight junction proteins, namely, claudin (CLDN)-c, -3 and -15 as well as occludin and zonula occludens-1, in the intestine of fish. In addition, Cu exposure increases the mRNA levels of the pro-inflammatory cytokines, specifically, IL-8, TNF-α and its related signalling factor (nuclear factor kappa B, NF-κB), which was partly correlated to the decreased mRNA levels of NF-κB inhibitor protein (IκB). These changes were associated with Cu-induced oxidative stress detected by corresponding decreases in glutathione (GSH) content, as well as decreases in the copper, zinc-superoxide dismutase (SOD1) and glutathione peroxidase (GPx) activities and mRNA levels, which were associated with the down-regulated antioxidant signalling factor NF-E2-related factor-2 (Nrf2) mRNA levels, and the Kelch-like-ECH-associated protein1 (Keap1) mRNA levels in the intestine of fish. Histidine supplementation in diets (3.7 up to 12.2 g/kg) blocked Cu-induced changes. These results indicated that Cu-induced decreases in intestinal TJ proteins and cytokine mRNA levels might be partially mediated by oxidative stress and are prevented by histidine supplementation in fish diet.

## Introduction

Heavy metals occur naturally in aquatic environments, and excess heavy metals are harmful to fish [1]. Copper (Cu) is a common heavy metal pollutant in aquatic environments that originates from a variety of anthropogenic sources, such as pesticides, fungicides and industrial wastes [2]. Intestines are organs that not only digest food and absorb nutrients but also provide a defensive barrier against ingested pathogens and noxious agents [3]. The intestine is one of the major target organs exposed to waterborne Cu in marine fish or dietary Cu in both marine and freshwater fish [4,5,6]. Our previous studies demonstrated that either waterborne Cu or dietary Cu exposure could cause lipid peroxidation and protein oxidation in the intestine of carps [7,8]. Al-Bairuty et al. also reported that waterborne Cu-exposure resulted in swollen goblet cells and villus tip erosion in the rainbow trout (*Oncorhynchus mykiss*) intestine [9]. These studies focused on heavy metal-induced intestinal histopathological changes in fish. However, to the best of our knowledge, few studies have investigated the mechanisms of heavy metal-induced intestinal damage in fish.

A previous study has demonstrated that the intestines provide a defence barrier against pathogens and noxious agents ingested in Atlantic salmon (*Salmo salar*) [10]. The intestinal barrier function is dependent on the expression of tight junction (TJ) proteins, including cytosolic one zonula occludens-1 (ZO-1) [11] and the transmembranal occludin (OCLD)[12] and claudins (CLDN) [13], which are ubiquitously expressed in metazoans [14] including fish [15]. The synthesis of OCLD is regulated by cytokines in Caco-2 cells [16]. Cytokines, in turn, are regulated by NF-κB, IκB and TOR signalling molecules in fish [17,18]. In mouse astrocytes, Öner et al. reported that Cu exposure could increase glucose content [19]. Glucose regulates the NF-κB signalling molecule in human umbilical artery smooth muscle cells [20]. Consistent with this finding, Cu exposure may affect cytokine production by modulating the relative number of signalling molecules to disrupt the structural integrity of the intestine in fish, which warrants investigation.

Disruptions of the integrity in fish intestine that are caused by Cu may be associated with an impaired intestinal antioxidant defence system [7]. Sandrini et al., (2011) reported that Cu exposure increases the reactive oxygen species (ROS) content in zebrafish hepatocytes [21]. ROS causes oxidative damage in fish erythrocytes [22]. Oxidative damage is closely related to the antioxidant defence system, which mainly consists of non-enzyme antioxidants, such as GSH and enzyme antioxidants, including SOD1 and GPx, in fish [23]. Cu exposure decreased SOD and GPx activities in the digestive gland of the pearl oyster (*Pinctada fucata*) [24]. The antioxidant enzyme activities are closely related to their gene expression levels in rats [25], which are regulated by the transcription factor NF-E2-related factor-2 (Nrf2); this regulation is inhibited by Kelch-like-ECH-associated protein 1 (Keap1) [26]. However, little attention has been given to the effects of Cu exposure on Nrf2 and Keap1 expression that impairs the intestinal antioxidant defence system in fish; further investigation is required.

Because Cu has potentially harmful effects in the intestine, it is important to prevent Cu toxicity-induced intestinal damage in fish. Several nutrients have been identified that prevent Cu toxicity, such as *myo*-inositol [7], α-tocopherol [27] and zinc [28]. Histidine is an important nutrient in fish [29]. It has a strong binding affinity for Cu ions that prevents Cu toxicity [30]. In addition, histidine has been reported to chelate copper in rainbow trout intestine [31,32,33]. Moreover, histidine is a scavenger of singlet oxygen via direct interactions with the imidazole ring [34]. Consistent with this finding, histidine may prevent Cu toxicity in fish intestine, which requires further investigations.

This study investigated the hypothesis that Cu damages the intestine of fish via changes in the intestinal TJ mRNA levels, in the intestinal antioxidant defence system and in the expression of histidine, which typically prevents Cu accumulation. This study is the first study to investigate Cu-induced toxicity on the TJ proteins, cytokines and Nrf2, Keap1, NF-κB, IκB and TOR signalling molecules in fish intestines. These results will provide understanding of Cu-induced intestinal damage mechanisms in fish. Moreover, this report is the first study to demonstrate that histidine prevents Cu-caused intestinal damage in fish, which may support the use of histidine as a Cu stress inhibitor and a potent antioxidant in an aquatic environment.

## Materials and Methods

### Ethics statement

Care of laboratory animals and animal experimentation were performed in accordance with animal ethics guidelines and approved by the Animal Ethics Committee of Sichuan Agricultural University. The committee defines the timing to give humane endpoints and the method of sacrifice. The criteria used in the present study to determine when the animals should be humanely sacrificed include the following activities: 1) Can the animal move? 2) Can the animal eat? and 3) What is the body temperature? Immediately after the animal is selected, we performed care for the maintenance of body temperature, moisture retention, nutrient supply and oxygen supply. Veterinary care was provided throughout conduction of the animal experiments. Fish were monitored daily during the study. If any clinical symptoms (i.e., morphological abnormality, restlessness or uncoordinated movements) were observed, then the fish were sedated by immersion in 2% benzocaine solution and subsequently euthanised by immersion in a 6% benzocaine solution (anaesthetic overdose) for 3 minutes to be certain that death was achieved. In addition, fish were transferred from the exposure cages to copper-free MS 222 metacaine containing fresh water and were subsequently sacrificed by a blow to the head. At the end of the trial, the animals were anaesthetised in a benzocaine solution and then humanely sacrificed.

### Preliminary test: Cu-induced oxidative stress in the fish intestine

CuSO_4_ was used in this study due to its extensive use as a pro-oxidant [35]. Cu exposure was performed according to the general Organization for Economic Co-operation and Development (OECD) guidelines for fish acute bioassays [36]. Clear water was used in this study for the low waterborne Cu concentration; this concentration, which was under the detection limit (0.002 mg/L), was measured using atomic absorption spectrometry. The water temperature and pH were maintained at 26 ± 2°C and 7.0 ± 0.5, respectively. The dissolved oxygen concentration was not less than 6.0 mg/L. A total of 180 young grass carp (initial average weight 558 ± 2.25 g) were randomly divided into 18 cages (1.4 × 1.4 × 1.4 m). In total, thirty fish were randomly assigned to each Cu concentration (0, 0.2, 0.7, 1.2, 1.7 and 2.2 mg Cu/L water) for 4 days, with 3 cages per concentration and 10 fish per cage. During Cu exposure, the fish were monitored every 6 h for health status. Mortality, if any, was recorded daily. The experiment was performed under a natural light and dark cycle. The fish were not fed.

### Growth trail

The basal diet composition is presented in Table 1. Fish meal, casein and gelatine were used as dietary protein sources for the low histidine diet. The dietary protein level was fixed at 300 g/kg diet, which was reported as optimum for the growth of young grass carp [37]. Crystalline amino acids (Jiangsu Nantong Eastern Amino Acid Co. Ltd., Nantong, China) were used to simulate the dietary amino acid profile to that of a young grass carp whole body of AA, excluding histidine, as previously described by Luo et al. (2014) [38]. L-histidine hydrochloride monohydrate was added to the basal diet to create six separate diets with serial doses of 0 (control), 2.0, 4.0, 6.0, 8.0 and 10.0 g histidine/kg diet. All of the experimental diet ingredients were mixed, pelleted and air dried according to Sevgili et al. [39]. Dry diets were stored at -20°C until use, according to the method described by Tan and Mai (2001) [40]. Histidine concentrations in the experimental diets were determined to be 2.0 (control), 3.7, 5.9, 7.9, 9.8 and 12.2 g/kg diet, according to the method described by Llames and Fontaine [41].

**Table 1:** Composition and nutrient content of the basal diet.

Ingredients	g/kg	Nutrients content^5^	g/kg
Fish meal	78.0	Crude protein	308.2
Casein	30.0	Crude lipid	46.8
Gelatin	39.9	Histidine	2.0
Amino acid premix^1^	199.9		
Histidine premix^2^	50.0		
Alpha-starch	280.0		
Corn starch	122.1		
Fish oil	22.0		
Soybean oil	18.9		
Vitamin premix^3^	10.0		
Mineral premix^4^	20.0		
Ca(H_2_PO_4_)_2_	22.7		
Choline chloride (500 g/kg)	6.0		
Cellulose	100.0		
Ethoxyquin(300 g/kg)	0.5		

^1^Amino acid mix (g/kg): arginine, 12.89 g; isoleucine, 12.69 g; leucine, 20.51 g; lysine, 17.13 g; methionine, 7.78 g; cystine, 0.91 g; phenylalanine, 13.60 g; tyrosine, 10.86 g; threonine, 11.88 g; tryptophan, 3.57 g; valine, 15.33 g; glutamic acid, 32.32 g; glycine, 40.40 g.

^2^ L-histidine hydrochloride monohydrate was added to obtain graded level of histidine. Each mixture was made isonitrogenous with the addition of reduced amounts of glycine and compensated with appropriate amounts of corn starch. Per kilogram of histidine premix composition from diet 1 to 6 was as follows (g/kg): L-histidine hydrochloride monohydrate 0.000, 54.89, 109.77, 164.66, 219.54 and 274.42 g; glycine 369.70, 311.11, 252.53, 193.94, 135.35 and 76.77 g; and corn starch 630.30, 634.00, 637.70, 641.40, 645.11 and 648.81 g, respectively.

^3^Per kilogram of vitamin premix (g/kg): retinyl acetate (500 000 IU g^-1^), 0.80 g; cholecalciferol (500,000 IU/g), 0.48 g; D, L-α-tocopherol acetate (500 g/kg), 20.00 g; menadione (230 g/kg), 0.22 g; cyanocobalamin (10 g/kg), 0.10 g; D-biotin (20 g/kg), 5.00 g; folic acid (960 g/kg), 0.52 g; thiamin hydrochloride (980 g/kg), 0.12 g; ascorhyl acetate (930 g/kg), 7.16 g; niacin (990 g/kg), 2.58 g; meso-inositol (990 g/kg), 52.33 g; calcium-D-pantothenate (900 g/kg) 2.78 g; riboflavine (800 g/kg), 0.99 g; pyridoxine hydrochloride (980 g/kg), 0.62 g. All ingredients were diluted with corn starch to 1 kg.

^4^Per kilogram of mineral premix (g/kg): FeSO_4_·H_2_O, 25.00 g; CuSO_4_·5H_2_O, 0.60 g; ZnSO_4_·H_2_O, 4.35 g; MnSO_4_·H_2_O, 2.04 g; KI, 1.10 g; NaSeO_3_, 2.50 g; MgSO_4_·H_2_O, 230.67 g. All ingredients were diluted with corn starch to 1 kg.

^5^Crude protein, crude lipid and histidine were measured value.

Fish management was followed according to the Guidelines for the Care and Use of Laboratory Animals of Sichuan Agricultural University as described by Wu et al. [42]. Grass carp were obtained from an aquaculture stock (Sichuan, China). All of the fish were acclimatised to the experimental conditions for 2 weeks. A total of 540 young grass carp (initial average weight 279.8 ± 1.07 g) were randomly allocated into 18 experimental cages (1.4 × 1.4 × 1.4 m) with 30 fish per cage. According to the method of Tang et al. [8], each cage was equipped with a disc that was 100 cm in diameter consisting of 1 mm gauze in the bottom to collect uneaten food. During the experimental period, each diet was distributed to triplicate groups of fish four times daily for 8 weeks, according to Du et al. (2006) [43]. After feeding for 30 minutes, uneaten feed was removed by siphoning as described by Lin et al. [44].

### Cu exposure

After the growth trial, 10 fish from each replicate were moved to labelled cages and exposed to 0.7 mg Cu/L water for 4 days (parameters used according to the preliminary test). Fish from the histidine deficiency group (Ctrl, 2.0 g histidine/kg diet) were in the cage without Cu for 4 days as the Ctrl/Ctrl treatment. In total, there were 7 different groups (pre-treatment histidine + Cu exposure). The fish were not fed, and experimental conditions were the same as those for the preliminary test.

### Sample collections and analysis

The fish from each cage were counted and weighed at the beginning and at the end of the growth trail to determine the percent weight gain (PWG), specific growth rate (SGR), feed efficiency (FE) and protein efficiency ratio (PER). At the end of the growth trail, blood from six fish per treatment group was drawn from the caudal vein six hours after the last feeding, and plasma was collected to determine the ammonia concentration as previously described by Tantikitti and Chimsung [45]. Fish were transferred from the exposure cages to copper-free MS 222 metacaine containing fresh water as described by Larsson et al. [46] and then humanely sacrificed according to Liu et al. [47]. The intestines were emptied of any leftover food and chyme by gently stroking the content out, and each segment was rinsed thoroughly in a phosphate-buffered saline (PBS) solution (145 mM NaCl, 8 mM Na_2_HPO_4_, 2 mM NaH_2_PO_4_, pH 7.2) according to the method described by Bakke et al. [48]. Intestinal segments were then frozen in liquid nitrogen and stored at −80°C until further analysis according to the method described by Wu et al. [49].

The intestinal malondialdehyde (MDA) and protein carbonyl (PC) contents were assessed as described by Livingstone et al. [50] and Armenteros et al. [51], respectively. The intestinal GSH content and SOD1 activities were assessed as described by McCarthy et al. [52], and the GPx activity was assessed as described by Ravn-Haren et al. [53]. The ammonia concentration was determined according to the method described by Tantikitti and Chimsung [45].

### RNA isolation and real-time quantitative PCR

Total RNA was extracted from the intestine using RNAiso Plus (Takara, Dalian, China) according to the manufacturer’s instructions, followed by DNase I treatment. Subsequently, total RNA quality and quantity were assessed by electrophoresis on 1% agarose gels and spectrophotometry, respectively. The absorption ratio of OD260/OD280 was between 1.9 and 2.1. cDNA was synthesised using a PrimeScript ^™^RT reagent Kit according to the manufacturer’s instructions (Takara, Dalian, Liaoning, China).

Specific primers for ZO-1 (KJ000055), claudin-12 (KF998571), TOR (JX854449), Nrf2 (KF733814), Keap1 (KF811013), NF-κB (KJ526214) and IκB (KJ125069) genes were designed for grass carp in our laboratory, and primers for claudin-b (KF193860), claudin-c (KF193859), claudin-3 (KF193858), claudin-15 (KF193857), occludin (KF193855), SOD1 (GU901214), GPx (EU828796), TNF-α (HQ696609), TGF-β (EU099588), IL-8 (JN663841), IL-10 (HQ388294) and β-actin (M25013) were designed using published grass carp sequences. To ensure that the primers are paralogue-specific for different isoforms, the primer pairs were designed in the poorly conserved regions of different isoforms of genes. Real-time PCR was performed for these genes according to standard protocols using the primers indicated in S1 Table. All real-time PCR reactions were performed on a CFX96^™^ Real-Time PCR Detection System (Bio-Rad, Laboratories, Inc.) using a SYBR^®^ Prime Script RT-PCR Kit II (Takara, Dalian, China). Amplicon was identified by sequencing. An additional dissociation curve analysis was performed, and it showed a single melting curve in all cases. No-RT controls were performed by omitting the addition of the reverse transcriptase enzyme, and no template controls were performed by the addition of nuclease free water. Several grass carp housekeeping genes, including 18S rRNA, β-actin and glycer-aldehyde-3-phosphate dehydrogenase (GAPDH), were tested as potential reference genes. Stability analysis by geNorm (version 3.5) software indicated that β-actin showed the greatest stability among groups (data not shown) and served as the reference gene. The thermocycling conditions were initiated with a denaturation step of 95°C for 30 s and then 40 cycles of PCR (denaturation at 95°C for 5 s, annealing for 30 s at a different temperature for each gene). Primer amplification efficiency was 100% for claudin-b, 100% for claudin-c, 100.1% for claudin-3, 99.9% for claudin-12, 100% for claudin-15, 100% for occludin, 100% for ZO-1, TOR, 99.8% for Nrf2, 100% for Keap1, 100% for NF-κB, 99.7% for IκB, 100% for SOD1, 100% for GPx, 100% for TNF-α, 99.8% for TGF-β, 100% for IL-8, 100% for IL-10 and 100% for β-actin. Expression results were analysed using the 2^-ΔΔCt^ method described by Livak and Schmittgen [54].

### Statistical analysis

The data are presented as the mean ± SD. A one-way analysis of variance (ANOVA) was applied to determine significant differences in the results of various groups over controls. *P*-values < 0.05 were considered significant. Dietary histidine requirements based on PWG and GPx were estimated using a quadratic regression analysis as described by Robbins et al. [55].

## Results

### Induction of oxidative stress in fish

**Fig 1:**
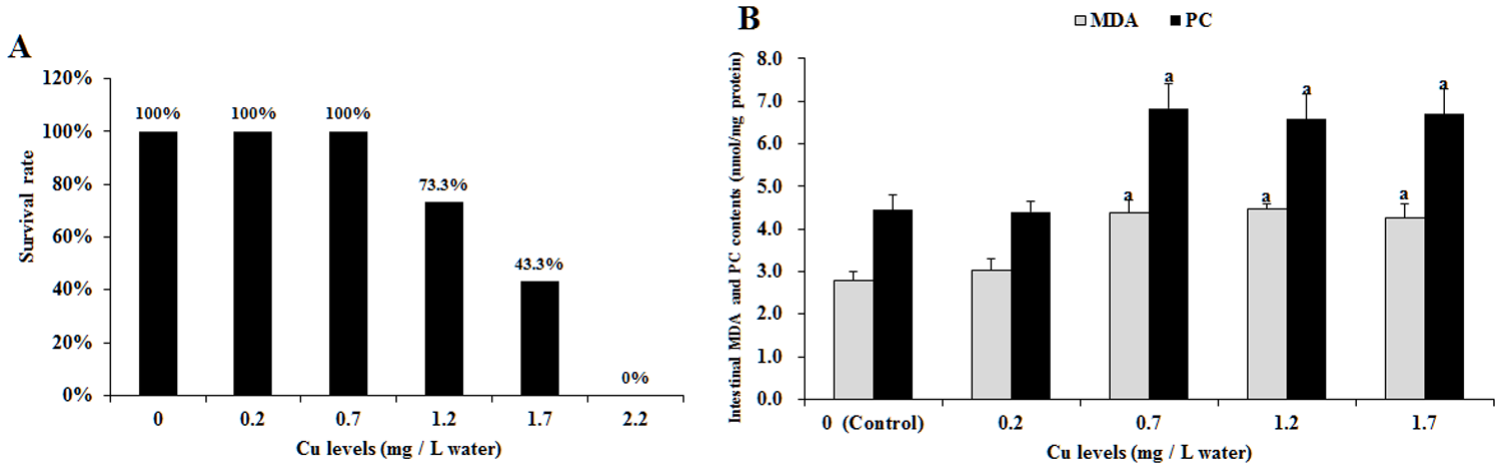
Cu induces oxidative stress in grass carp intestine. (A) The survival rate of young grass carp following copper exposure for 4 days. (B) The entire intestinal MDA and PC content of young grass carp following Cu exposure for 4 days. Values are expressed as the mean with standard deviation and are represented by vertical bars (n = 6). Superscript (^a^) indicates a significant (*P* < 0.05) difference over control values. MDA, malondialdehyde; PC, protein carbonyls.

The survival rate and intestinal MDA and PC contents of the fish following Cu exposure are shown in Fig 1. These results indicated that the survival rate of fish gradually decreased with increasing Cu levels (Fig 1A). The intestinal MDA and PC contents were higher in fish that had been exposed to 0.7, 1.2 and 1.7 mg Cu/L water than the unexposed Ctrl and those contents that were exposed to 0.2 mg Cu/L water fish (Fig 1B).

### Growth performance parameters and plasma ammonia concentrate (PAC)

**Fig 2:**
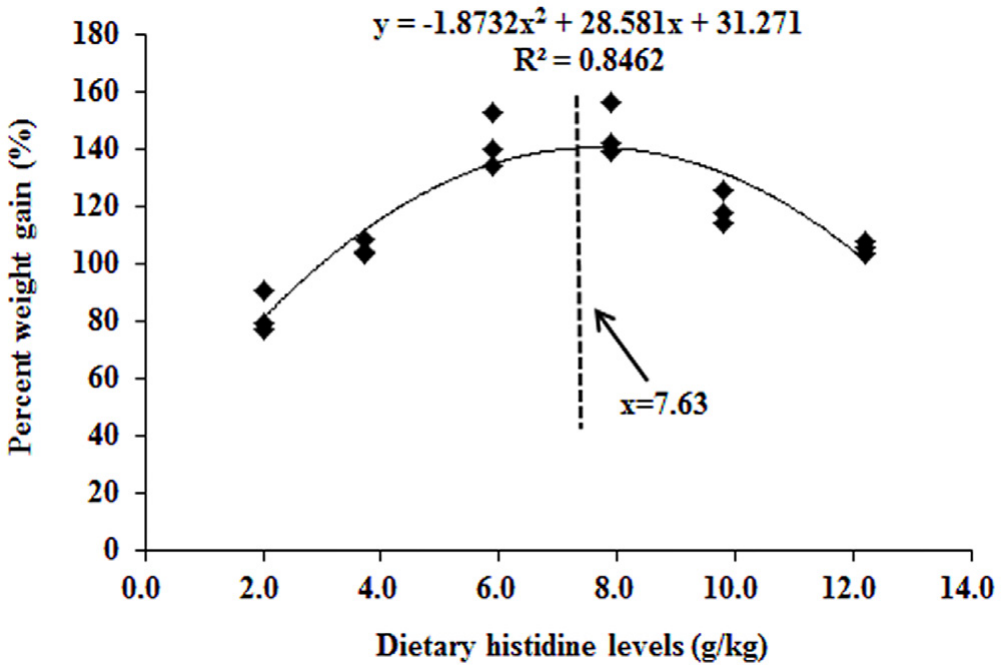
Quadratic regression of the percentage of weight gain of grass carps as a function of histidine dose.

The effects of dietary histidine on growth performances and PAC are presented in Table 2. PWG, SGR, FE, PER and FI were the lowest in fish that had been fed with the histidine-unsupplemented basal diet, and the values increased with increasing dietary histidine levels up to 7.9 mg/kg diet (PWG, SGR, FE and PER) and 5.9 mg/kg diet (FI); the levels decreased with additional increases in histidine concentrations. PAC was significantly decreased with dietary histidine levels up to 7.9 g/kg diet. The dietary histidine requirement of young grass carp (279.1–685.4 g) as estimated using a quadratic regression analysis based on the PWG (Fig 2) was 7.63 g/kg, which corresponded to 24.76 g/kg dietary protein.

**Table 2:** Growth performance and plasma ammonia concentration^1^.

Histidine^1^	Control	3.7	5.9	7.9	9.8	12.2
IBW^2^	280.6 ± 0.84	279.6 ± 1.68	280.5 ± 0.69	279.1 ± 1.02	279.3 ± 0.67	279.4 ± 0.73
FBW^2^	511.2 ± 19.0	573.1 ± 8.34^a^	679.3 ± 26.41^a^	685.4 ± 26.3^a^	612.4 ± 18.0^a^	574.2 ± 6.94^a^
PWG^2^	82.2 ± 7.24	105.0 ± 2.71^a^	142.2 ± 9.69^a^	145.6 ± 9.09^a^	119.2 ± 5.92^a^	105.6 ± 2.16^a^
SGR^2^	1.07 ± 0.07	1.28 ± 0.02^a^	1.58 ± 0.07^a^	1.60 ± 0.05^a^	1.40 ± 0.05^a^	1.29 ± 0.02^a^
FI^2^	445.1 ± 5.00	498.5 ± 3.06^a^	627.5 ± 2.15^a^	602.7 ± 2.53^a^	548.3 ± 1.53^a^	499.7 ± 3.06^a^
FE^2^	51.80 ± 3.87	58.89 ± 1.92^a^	63.51 ± 4.44^a^	67.41 ± 4.04^a^	60.75 ± 3.1^a^	59.01 ± 0.93^a^
PER^2^	1.66 ± 0.12	1.89 ± 0.04^a^	2.04 ± 0.14^a^	2.16 ± 0.13^a^	1.95 ± 0.10^a^	1.89 ± 0.03^a^
PAC^3^	365.3 ± 10.8	262.3 ± 12.9^a^	176.8 ±11.2^a^	153.6 ± 7.32^a^	249.3 ± 11.1^a^	344.2 ± 12.3^a^
Regressions						
Y_PWG_ = -1.965x^2^ + 29.602x + 29.111			R^2^ = 0.9094		*P* < 0.05	
Y_SGR_ = -1.5893x^2^ + 1.8021x + 1.05			R^2^ = 0.9209		*P* < 0.05	
Y_FI_ = -5.6275x^2^ + 83.82x + 292.91			R^2^ = 0.8800		*P* < 0.05	
Y_FE_ = -0.004x^2^ + 0.0614x + 0.4159			R^2^ = 0.9189		*P* < 0.05	
Y_PER_ = -0.0129x^2^ + 0.1995x + 1.3212			R^2^ = 0.9046		*P* < 0.05	
Y_PAC_ = 7.6694x^2^–108.79x + 553.63			R^2^ = 0.9692		*P* < 0.05	

Superscript (^a^) in the same row indicates a significant (*P* < 0.05) difference over control values.

^1^Upper half of the table shows different parameters of growth performance (see below) of young grass carp fed diets with graded levels of histidine (first row, g/kg). The lower half shows the regression analysis of data in the upper half of the table.

^2^Values are expressed as the mean ± SD, mean of three replicates with thirty fish per replicate. The mean values within the same row with different superscripts are significantly different (*P* < 0.05).

^3^Plasma ammonia concentration (PAC, μmol/L). Values are expressed as the mean ± SD (n = 6), and mean values within the same row with different superscripts are significantly different (*P* < 0.05).

IBW: initial body weight (g/fish); FBW: final body weight (g/fish); PWG: percentage weight gain (%); SGR: specific growth rate (%/day); FI: feed intake (g/fish); FE: feed efficiency (%); PER: protein efficiency ratio; PAC: plasma ammonia content.

Weight gain (WG) = FBW (g)–IBW (g);

PWG = 100 × WG (g)/IBW (g);

SGR = 100 × [ln FBW (g)-ln IBW (g)]/number of days;

FE = 100 × weight gain (g)/feed intake (g);

PER = wet weight gain (g)/protein intake (g);

### Claudin family, occludin and ZO-1 mRNA levels in the entire intestine after exposure to 0.7 mg Cu/L water for 4 days

**Fig 3:**
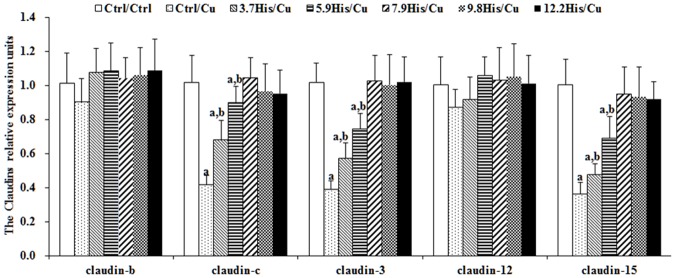
The claudin mRNA levels in the entire intestine as a function of histidine. Young grass carps were fed for 8 weeks with diets with different doses of histidine, and then exposed to 0.7 mg Cu/L water for 4 additional days. Values are expressed as the mean with standard deviation and are represented by vertical bars (n = 6). Superscript (^a^) indicates a significant (*P* < 0.05) difference over Ctrl/Ctrl values. Superscript (^a,b^) indicates a significant (*P* < 0.05) difference over Ctrl/Ctrl and Ctrl/Cu values.

**Fig 4:**
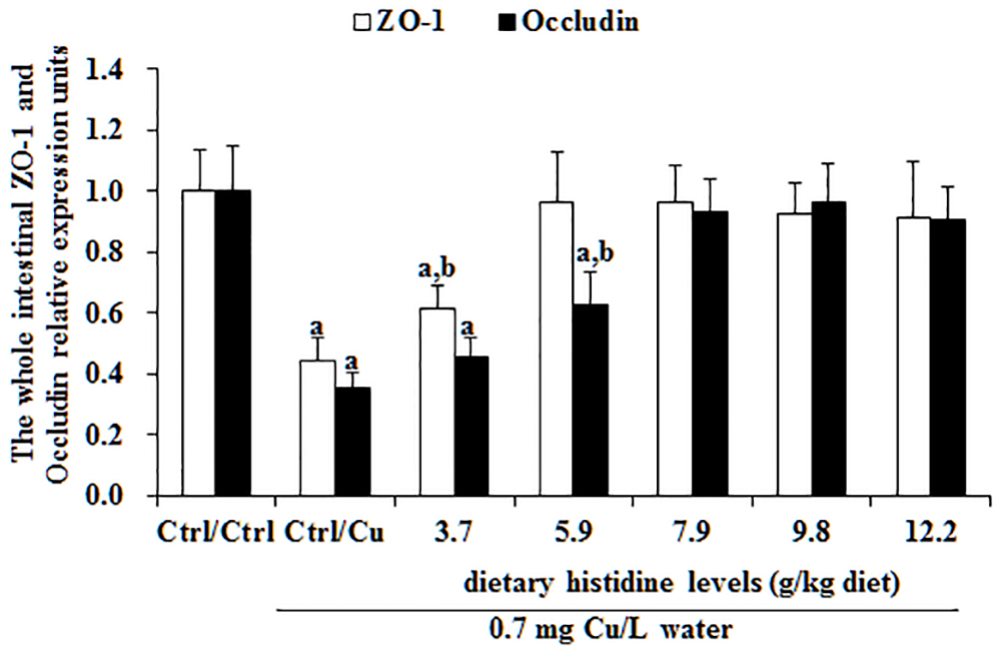
The ZO-1 and occludin mRNA levels in entire intestines of grass carp fed with different histidine doses. The protocol and indications are shown in Fig 3.

Cu toxicity as well as its prevention by dietary histidine on claudin-b, claudin-c, claudin-3, claudin-12, claudin-15, ZO-1 and occludin mRNA levels in the entire intestine are presented in Figs 3 and 4. These results indicate that compared with the Ctrl/Ctrl group, the Ctrl/Cu treatment group significantly down-regulated claudin-c (Fig 3), claudin-3 (Fig 3), claudin-15 (Fig 3), occludin (Fig 4) and ZO-1 (Fig 4) mRNA levels. However, histidine pre-supplementation significantly prevented changes in claudin-c, claudin-3, claudin-15 and ZO-1 mRNA levels. Claudin-b and claudin-12 mRNA levels were not significantly affected by Cu exposure or histidine pre-supplementation (Fig 3).

### The mRNA levels of cytokines, NF-κB, IκB and TOR in three intestinal segments after exposure to 0.7 mg Cu/L water for 4 days

**Fig 5:**
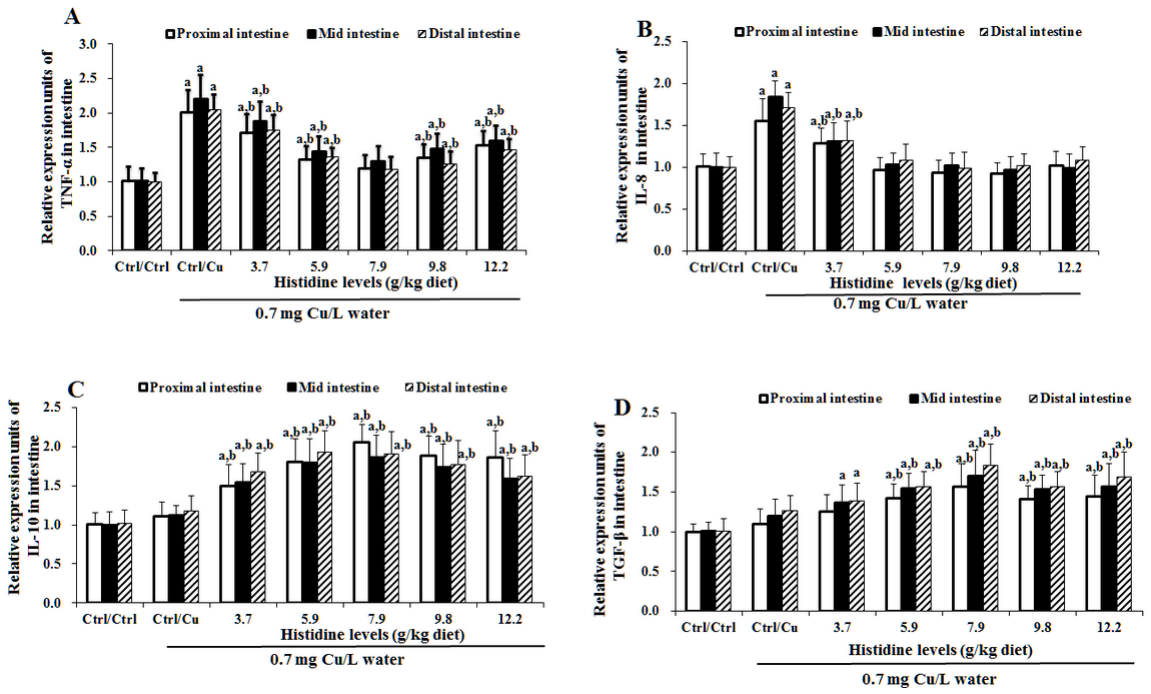
Cu induces increases in cytokine mRNA levels in carps. The (A) TNF-α, (B) IL-8, (C) IL-10, and (D) TGF-β mRNA levels in proximal intestine, mid intestine and distal intestine of young grass carp. The protocol and indications are shown in Fig 3. TNF-α, Tumour necrosis factor α; IL-8, interleukin 8; IL-10, interleukin 10; TGF-β, transforming growth factor β.

**Fig 6:**
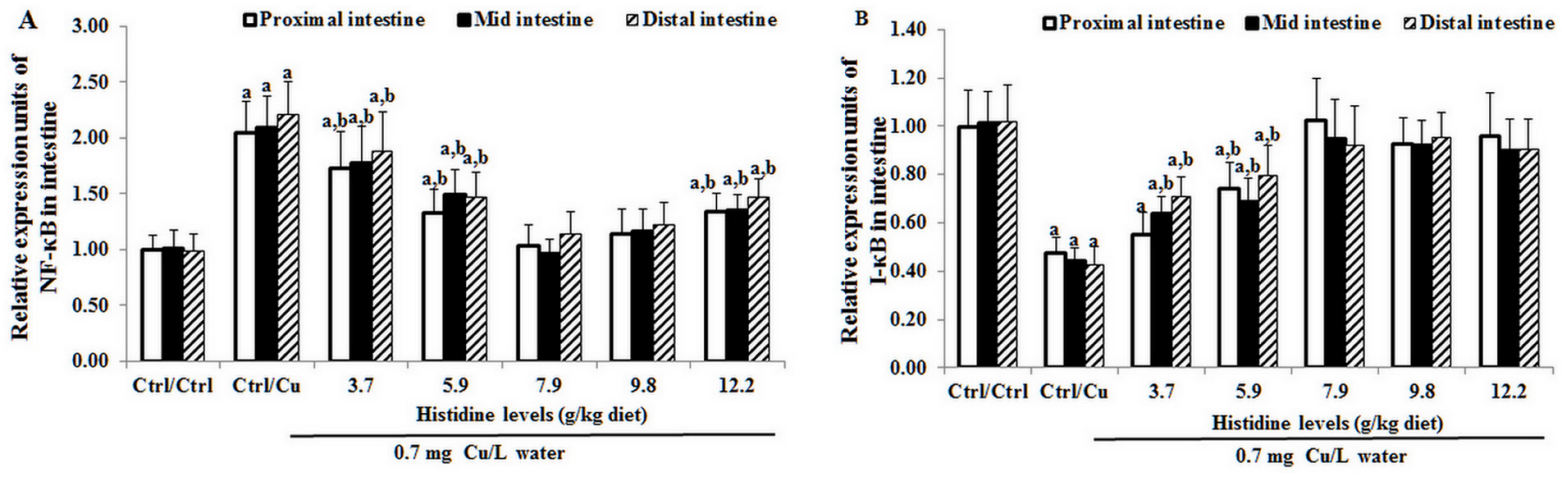
Cu induces an increase in NF-κB (A) and IκB (B) mRNA levels in fish intestine. The protocol and indications are shown in Fig 3. NF-κB, nuclear factor kappa B; IκB, inhibitor protein-κB.

**Fig 7:**
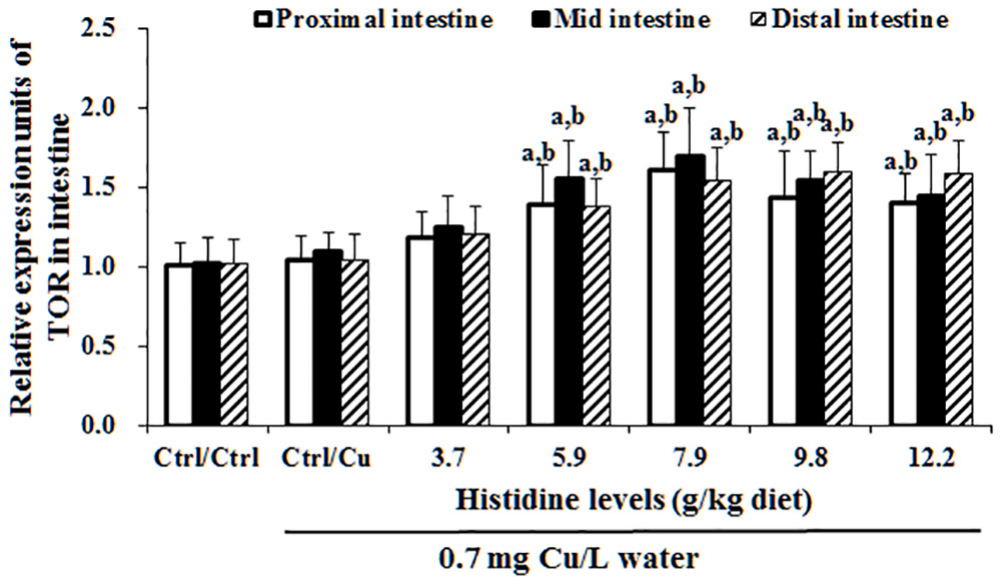
Cu induces an increase in TOR mRNA levels in fish intestine. Protocol and indications are shown in Fig 3. TOR, Target of rapamycin.

Cu toxicity as well as its prevention by dietary histidine on TNF-α, IL-8, TGF-β, IL-10 and TOR mRNA levels in the PI, MI and DI are presented in Figs 5–7. The results indicated that compared with the Ctrl/Ctrl group, Ctrl/Cu treatment significantly up-regulated TNF-α (Fig 5A), IL-8 (Fig 5B) and NF-κB (Fig 6A) mRNA levels in the PI, MI and DI of fish. Expectedly, histidine pre-supplementation completely prevented these changes in the PI, MI and DI. Ctrl/Cu treatment down-regulated IκB mRNA levels in the intestine, which were completely prevented by histidine pre-supplementation (Fig 6B). The IL-10 (Fig 5C), TGF-β (Fig 5D) and TOR (Fig 7) mRNA levels were not significantly affected by Cu exposure. However, histidine pre-supplementation significantly up-regulated IL-10, TGF-β and TOR mRNA levels in the PI, MI and DI.

### Antioxidant-related parameters in the intestine after exposure to 0.7 mg Cu/L water for 4 days

**Fig 8:**
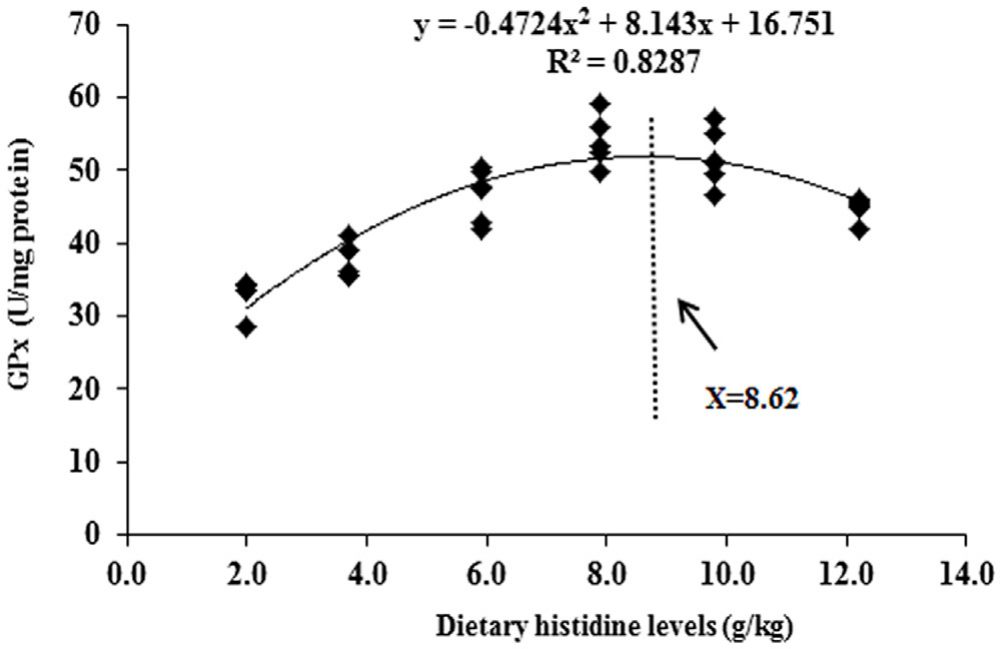
A quadratic regression analysis of the entire intestinal glutathione peroxidase activity for young grass carp fed with diets containing graded levels of histidine for 8 weeks, followed by exposure to 0.7 mg Cu/L water for 4 days.

**Fig 9:**
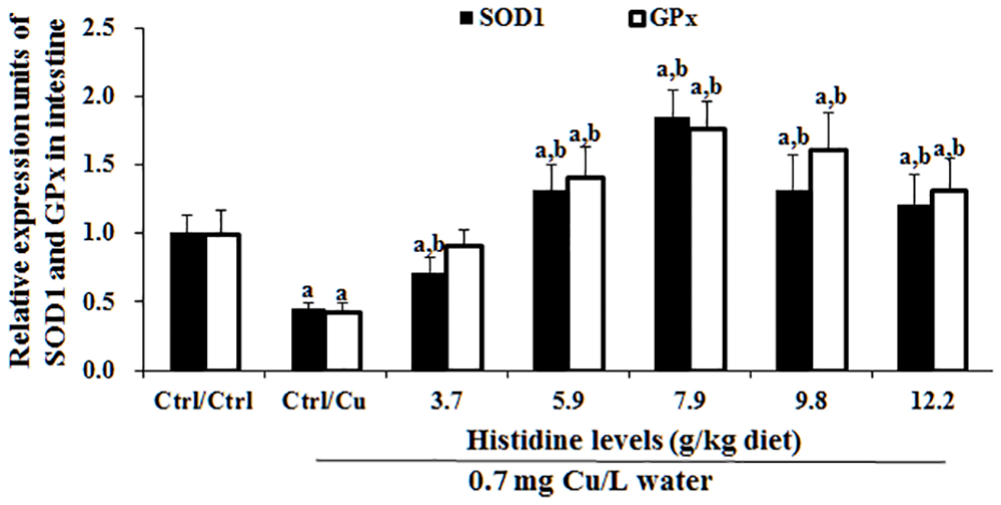
Cu induces increases in SOD1 and GPx mRNA levels in the entire intestine of grass carp. The protocol and indications are shown in Fig 3. SOD1, copper, zinc-superoxide dismutase; GPx, glutathione peroxidase.

**Fig 10:**
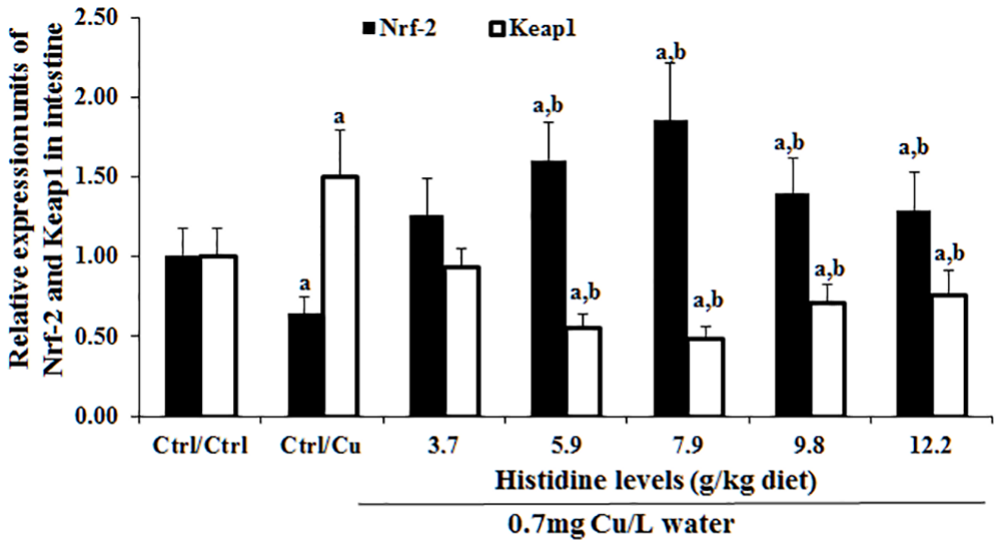
Cu induces increases in Nrf2 and Keap1 mRNA levels in the entire intestine of grass carp. The protocol and indications are shown in Fig 3. Nrf2, NF-E2-related factor-2; Keap1, Kelch-like-ECH-associated protein.

The intestinal MDA, PC and GSH contents as well as SOD1 and GPx activities are presented in Table 3. These results indicated that compared with the Ctrl/Ctrl group, Ctrl/Cu treatment significantly increased the MDA and PC contents and decreased the SOD and GPx activities and the GSH content. However, histidine pre-supplementation prevented these changes in the intestine. The dietary histidine requirement of young grass carp (279.1–685.4 g), as estimated using a quadratic regression analysis based on GPx (Fig 8), was 8.62 g/kg diet, which corresponded to 27.97 g/kg dietary protein. Cu exposure and prevention by histidine on the SOD1, GPx, Nrf2 and Keap1 mRNA levels are presented in Figs 9 and 10. These results indicated that compared with the Ctrl/Ctrl group, Ctrl/Cu treatment significantly down-regulated SOD1 and the GPx levels and up-regulated the Keap1 mRNA levels (Fig 10). Expectedly, histidine pre-supplementation prevented these changes in the intestine (Fig 10).

**Table 3:** Cu-induced oxidative stress can be prevented by histidine.

Histidine	MDA	PC	SOD1	GPX	GSH
Ctrl/Ctrl	2.19±0.18	4.64±0.26	4.70±0.34	48.15±2.71	6.19±0.48
Ctrl/Cu	4.26±0.24^a^	7.83±0.66^a^	2.16±0.17^a^	33.09±2.29^a^	2.95±0.23^a^
3.7 (g/kg diet)/ Cu	3.38±0.19^a,b^	6.63±0.32^a,b^	2.85±0.24 ^a,b^	46.72±3.00	5.20±0.37 ^a,b^
5.9 (g/kg diet) / Cu	2.68±0.12^a,b^	4.47±0.39	4.16±0.29 ^a,b^	55.70±5.20 ^a,b^	6.37±0.48
7.9 (g/kg diet) / Cu	2.36±0.15	4.26±0.28	6.42±0.38 ^a,b^	54.25±3.22 ^a,b^	6.96±0.56 ^a,b^
9.8 (g/kg diet) / Cu	3.13±0.11 ^a,b^	4.70±0.34	6.01±0.24 ^a,b^	45.49±2.68	5.64±0.47 ^a,b^
12.2 (g/kg diet) / Cu	3.19±0.16 ^a,b^	5.62±0.48 ^a,b^	4.94±0.40	44.88±1.51	4.20±0.35 ^a,b^

Superscript (^a^) in the same column indicates a significant (*P* < 0.05) difference over Ctrl/Ctrl values. Superscript (^a,b^) in the same column indicates a significant (*P* < 0.05) difference over Ctrl/Ctrl and Ctrl/Cu values. Malondialdehyde content (MDA, nmol/mg protein), protein carbonyl content (PC, nmol/mg protein), copper, zinc-superoxide dismutase (SOD1, U/mg protein), glutathione peroxidase (GPx, U/mg protein) activities and glutathione (GSH, mg/g protein). Values expressed as the mean ± SD, n = 6. Mean values in the same column with different superscripts are significantly different (*P* < 0.05).

## Discussion

A growth trial is a method of nutrient deposition that is used to investigate the prevention of toxicity mediated by specific nutrients [56]. In this study, the deficiency of histidine decreased PWG, FI, FE and PER, whereas this amino acid supplementation improved these values in young grass carp. These results indicated that we performed a successful growth trial to lay the foundation for further studies.

Oxidative stress is commonly assessed by MDA and PC formation [56]. We demonstrated that Cu (0.7 mg/L, 4 days) is a sublethal concentration that induces oxidative stress in the intestine of fish and allows the investigation of whether Cu toxicity can be prevented by histidine. However, how waterborne Cu reaches the intestine in freshwater fish remained unclear because this fish does not drink, and any drinking is minimal. It is well known that gills are the first organs that come into contact with environmental pollutants in marine and freshwater fish [57]. Kamunde et al. reported that Cu absorbed from the water via gills is likely transported via arterial circulation and deposited into other peripheral tissues before reaching the liver, and after 48 h, newly accumulated Cu concentration in the tissues were ranked in order as liver > gill > gut > plasma > carcass in rainbow trout [58]. Thus, we assumed that intestinal Cu in freshwater fish under waterborne exposure might be accumulating mainly from gill absorption and arterial circulation, which requires further investigations.

### Cu changes TJ proteins, cytokines and NF-κB, IκB and TOR signalling molecules in the fish intestine

Herpes 3 infection induces a decrease in CLDN-3 mRNA in fish [59] similar to the one shown in this study. In general, a reduction in mRNA levels is associated with a corresponding reduction in protein and a disruption in its function. Nevertheless, tight junction disruption, which is associated with protein reduction, is sometimes accompanied by an increase, rather than a decrease, in the corresponding mRNA. Thus, the reduction in CLDN-2 that is caused by the epidermal growth factor [60], several tight junction proteins by ouabain [61] and ZO-1 in subconfluent cells [62] in Madin-Darby canine kidney cells resulted in a disruption in tight junctions and an increase in mRNA levels corresponding to the mentioned proteins. However, this does not appear to be the case with a Cu-induced decrease in CLDNs as observed in this study because carps showed a decrease in their survival rate, which may result, in part, from the loss of intestinal tight junctions. The results of this study demonstrated that compared with the Ctrl/Ctrl group, Ctrl/Cu treatment down-regulated the claudin-c, claudin-3, claudin-15, occludin and ZO-1 mRNA levels in the intestine of grass carp. To the best of our knowledge, this is the first report regarding the effect of Cu exposure on the TJ protein mRNA levels in fish. Several previous studies have demonstrated that TNF-α treatment decreased the ZO-1 protein levels in Caco-2 cells [63], the occludin protein levels in mouse astrocytes [64] and the claudin-3 mRNA levels in mouse ilea [65]. In addition, IL-8 treatment decreases the ZO-1 and occludin mRNA levels in human vascular endothelial cells [66]. The present study demonstrated that compared with the Ctrl/Ctrl group, Ctrl/Cu treatment up-regulated the TNF-α and IL-8 mRNA levels in the intestine of grass carp, which was in contrast to changes in claudin-3, occludin and ZO-1, suggesting that Cu-induced decreases in these TJ mRNA levels, at least in part due to the up-regulation of TNF-α and IL-8 mRNA in the intestine of fish. In addition, TGF-β treatment decreased the claudin-12 mRNA levels in human oral epithelial cells [67]. Interestingly, in this study, compared with the Ctrl/Ctrl group, the Ctrl/Cu treatment did not change the TGF-β mRNA levels in the intestine of grass carp, which was similar to a change in claudin-12, indicating that claudin-12 is partly regulated by TGF-β in the fish intestine. However, there is no information regarding the effect of Cu exposure on cytokines in fish intestine. Cytokine production is regulated by signalling molecules in mammals [68]. Thus, we next investigated the effects of Cu on the signalling molecules in the intestine of fish.

NF-κB regulates a number of target genes, including TNF-α and IL-8 in fish [17]. The present study demonstrated that compared with the Ctrl/Ctrl group, Ctrl/Cu treatment increases the intestinal NF-κB mRNA levels in grass carp, which were similar to the changes observed in the TNF-α and IL-8 mRNA levels, indicating that Cu exposure-increased the TNF-α and IL-8 mRNA levels that might be partially related to the increase in the NF-κB mRNA levels in the intestine of fish. In fish, NF-κB is directly inhibited by the NF-κB inhibitor protein (IκB) [69]. Beg et al. reported that down-regulation of IκB gene expression up-regulated the NF-κB mRNA levels in mice [70]. In the present study, compared with the Ctrl/Ctrl group, the Ctrl/Cu treatment decreased the IκB mRNA levels in the intestine of grass carp, suggesting that Cu-induced NF-κB mRNA increases might be partially related to the decreased IκB mRNA levels in the fish intestine. Anti-inflammatory cytokines, such as IL-10 [71] and TGF-β [18] are up-regulated by the TOR signalling molecule in mammals. Interestingly, the present study demonstrated that compared with the Ctrl/Ctrl group, Ctrl/Cu treatment did not change the TOR mRNA levels in the intestine of grass carp, which were similar to the changes observed in the IL-10 and TGF-β mRNA levels, indicating that the unchanged IL-10 and TGF-β mRNA levels might be caused by TOR signalling in the intestine of fish. A potential explanation is the different sensitivity of TOR to external stimuli. The TOR signalling molecule is sensitive to nutrient and insulin stimuli in vertebrates [72]. In addition, Cu-induced changes in the intestinal TJ mRNA levels might be partially associated with an impaired intestinal antioxidant defence system [7]. Thus, we next investigated the effects of Cu on the intestinal antioxidant defence system.

### Cu impaired the intestinal antioxidant defence system by affecting signalling molecules Nrf2 and Keap1 in fish intestine

Cu is an important pollution-causing metal [73]. Cu exposure could induce oxidative damage in the pacu (*Piaractus mesopotamicus*) liver [74]. In the current study, compared with the Ctrl/Ctrl group, Ctrl/Cu treatment increased MDA and PC content by approximately 95% and 69%, respectively, decreased SOD1 and GPx activity by approximately 118% and 46%, respectively, and decreased GSH content by approximately 110% in the intestine of young grass carp, suggesting that Cu induces severe antioxidant defence system injury in fish intestine. These changes were similar to those observed by Jiang et al. [7]. Enzyme activities were also highly related to the mRNA levels in animals [75]. In the present study, compared with Ctrl/Ctrl group, Ctrl/Cu treatment decreased the SOD1 and GPx mRNA levels, which were similar to their enzyme activities, suggesting that Cu-inhibited SOD1 and GPx activities might be partially related to the down-regulation of their corresponding mRNA levels in the intestine of fish. The antioxidant enzyme mRNA levels are regulated by the signalling pathway-related molecules in vertebrates [26]. Nrf2, a master signalling molecule of the endogenous antioxidant defence system, is a critical regulator of antioxidant enzyme gene expression in mice [76]. In the present study, compared with the Ctrl/Ctrl group, Ctrl/Cu treatment down-regulated the Nrf2 mRNA levels, which was similar to the changes in the SOD1 and GPx mRNA levels, suggesting that Cu-caused down-regulation of SOD1 and GPx mRNA levels might be partially related to the down-regulation of Nrf2 mRNA in the intestine of fish. In addition, up-regulation of antioxidant enzyme gene transcription by Nrf2 is largely dependent on its nuclear translocation [77]. Keap1 is an inhibitor of Nrf2, which binds Nrf2 to prevent its nuclear translocation [78], and Keap1 dissociation from Nrf2/Keap1 enhances Nrf2 nuclear translocation [79]. It has been reported that up-regulation of Keap1 gene expression inhibits Nrf2 nuclear translocation in human bile duct cancer cells [80]. In this study, compared with the Ctrl/Ctrl group, Ctrl/Cu treatment up-regulates the Keap1 mRNA levels, suggesting that Cu exposure down-regulated the Nrf2 mRNA levels and might be partially correlated to the inhibition of Nrf2 nuclear translocation in the intestine of fish. Overall, the present study has demonstrated Cu-mediated toxicity on TJ proteins, cytokines and the antioxidant defence system in the fish intestine. Thus, it is necessary to expand our knowledge to prevent these types of toxicity from accumulating in fish intestines.

### Histidine prevented Cu-induced damage in the intestine of fish

Histidine is an important amino acid in fish [29]. Our results indicated that histidine deficiency significantly increases MDA and PC content, decreases SOD1 and GPx activities and GSH content, and also significantly down-regulates the claudin-c, claudin-3, claudin-15, occludin, ZO-1, IL-10, TGF-β, IκB, TOR, SOD1, GPx, Nrf2 mRNA levels and up-regulates the IL-8, TNF-α, NF-κB and Keap1 mRNA levels, whereas optimal histidine pre-supplementation completely restored these changes in the intestine of fish. These results indicate that histidine can prevent Cu exposure-induced changes in tight junction proteins, cytokines and the antioxidant defence system in the intestine of fish. Taken together, these results may identify measures to ameliorate metal toxicity in fish. Histidine-prevented Cu toxicity in fish might be partially related to the chelation of Cu. Our previous study observed that chelating Cu by *myo*-inositol could reduce the toxic effect of Cu at the intestine/enterocytes in Jian carp [7,81]. Histidine could chelate copper in rainbow trout intestine [31,32,33]. Moreover, chelating of Cu by nutrients was also reported by Bopp et al. (2008), who observed that the addition 5 μM CuSO_4_ corresponded to a free Cu^2+^ concentration of 0.11 μM in Earle’s medium [73]. All of these observations support our hypothesis. Interestingly, after Cu exposure, the excess histidine pre-supplementation groups demonstrated decreased GSH content and SOD1 and GPx activities compared with the optimal pre-supplementation groups. One potential explanation is the production of ROS by excessive histidine. Histamine, the metabolite of histidine, is abundantly produced by excess histidine, resulting in increased ROS production in breast cancer cells [82]. ROS can also decrease the SOD1 and GPx activities and the GSH content in carp erythrocytes [22]. Excessive histidine pre-supplementation demonstrated harmful effects on preventing Cu toxicity in the intestine of fish. Thus, we must determine the precise histidine requirement to prevent Cu toxicity in young grass carp. The nutritional requirement is determined by sensitive biomarkers in animals [83]. Several studies in fish indicated that GPx [84] and PWG [85] are sensitive indictors of heavy metal detoxification and growth performance, respectively. In the present study, the histidine requirements for grass carp (279.1–685.4 g) were 8.62 and 7.63 g/kg diet or 27.97 and 24.76 g/kg dietary protein based on the GPx and PWG, respectively. Interestingly, the histidine requirement for preventing Cu toxicity was greater than for growth performance. This phenomenon may be due to the presence of histidine in the active centres of numerous enzymes in fish and its highly attack by heavy metal ions. Thus, a higher histidine level ensures enzyme activities under heavy metal exposure conditions.

## Conclusions

**Fig 11:**
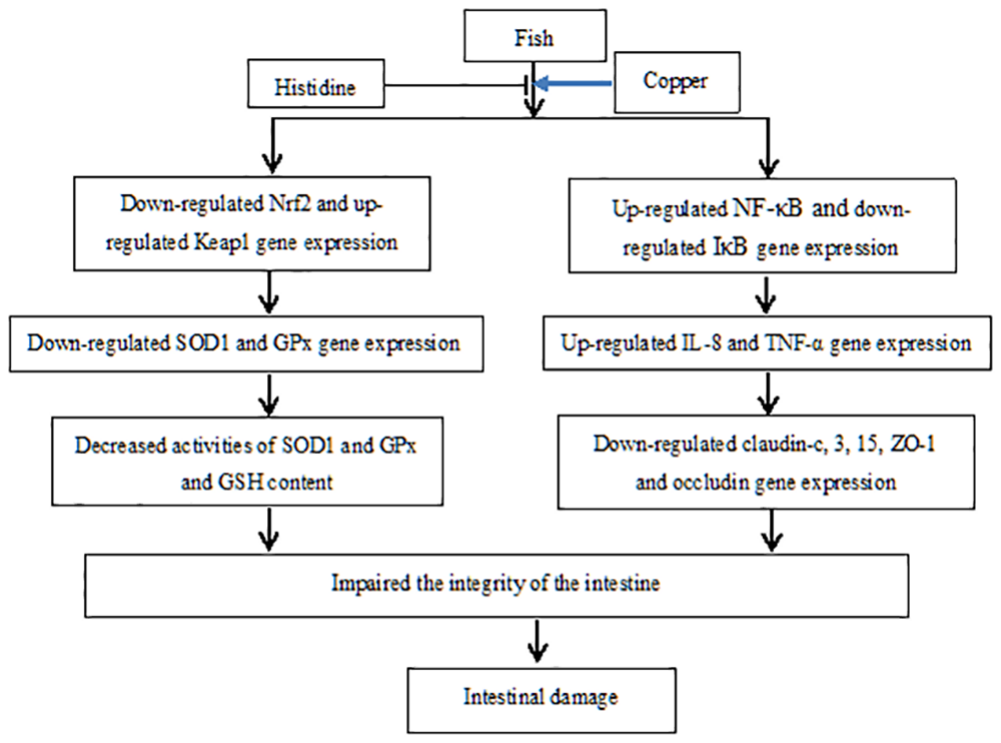
The proposed action pathway of Cu-induced damage of fish intestine.

We are the first to report that Cu exposure induces a decrease in the expression of genes that encode the intestinal TJ proteins CLDN-c, CLDN-3 and CLDN-15, as well as occludin and ZO-1, but neither CLDN-b nor CLDN-12 in the fish intestine. These changes may result, at least partially, from increases in the cytokines TNF-α and IL-8, but not in TGF-β and IL-10 and from Cu-induced oxidative stress. We also demonstrated that changes in cytokines and antioxidant enzymes might be correlated to the signalling molecules NF-κB, IκB, Nrf2 and Keap1 (Fig 11). Histidine supplemented in the diets (3.7 up to 12.2 g/kg) blocks Cu-induced changes. These results provide further understanding of how Cu induces intestinal damage and demonstrate that histidine effectively prevents Cu toxicity in fish.

## Supporting Information

S1 Table Real-time primer sequences, annealing temperature and amplicon for zonula occludens-1 (ZO-1), Occludin, Claudin-b, Claudin-c, Claudin-3, Claudin-12, Claudin-15, tumour necrosis α (TNF-α), interleukin 8 (IL-8), interleukin 10 (IL-10), transforming growth factor β (TGF-β), Nuclear factor kappa B (NF-κB) and inhibitor protein-κB (IκB), target of rapamycin (TOR), copper, zinc-superoxide dismutase (SOD1), glutathione peroxidase (GPx), NF-E2-related factor-2 (Nrf2) and Kelch-like-ECH-associated protein1 (Keap1) and β-Actin genes. (DOCX)
