# Supplementary material for: Histidine Prevents Cu-Induced Oxidative Stress and the Associated Decreases in mRNA from Encoding Tight Junction Proteins in the Intestine of Grass Carp (Ctenopharyngodon idella)
Source: PLoS One. 2016 Jun 9;11(6):e0157001. doi: 10.1371/journal.pone.0157001 (PMC4900568; doi:10.1371/journal.pone.0157001)
Supplement: S1 Table — (DOCX) [file pone.0157001.s001.docx]

**Supplemental information**

**S1 Table**

| Gene | Primerssequences  forward | Primerssequences  revers | Annealing temperature | Amplicon (bp) |
| --- | --- | --- | --- | --- |
| ZO-1 | 5^′^-CGGTGTCTTCGTAGTCGG-3^′^ | 5^′^-CAGTTGGTTTGGGTTTCAG-3^′^ | 59.4°C | 154 |
| Occludin | 5^′^-TATCTGTATCACTACTGCGTCG-3^′^ | 5^′^-CATTCACCCAATCCTCCA-3^′^ | 59.4°C | 208 |
| Claudin-b | 5^′^-GAGGGAATCTGGATGAGC-3^′^ | 5^′^-ATGGCAATGATGGTGAGA-3^′^ | 57.0°C | 122 |
| Claudin-c | 5^′^-GAGGGAATCTGGATGAGC-3^′^ | 5^′^-CTGTTATGAAAGCGGCAC-3^′^ | 59.4°C | 241 |
| Claudin-3 | 5^′^- ATCACTCGGGACTTCTA-3^′^ | 5^′^- CAGCAAACCCAATGTAG-3^′^ | 57.0°C | 82 |
| Claudin-12 | 5^′^-CCCTGAAGTGCCCACAA-3^′^ | 5^′^-GCGTATGTCACGGGAGAA-3^′^ | 55.4°C | 81 |
| Claudin-15 | 5^′^-TGCTTTATTTCTTGGCTTTC-3^′^ | 5^′^-CTCGTACAGGGTTGAGGTG-3^′^ | 59.0°C | 115 |
| TNF-α | 5^′^-CGCTGCTGTCTGCTTCAC-3^′^ | 5^′^-CCTGGTCCTGGTTCACTC-3^′^ | 58.4°C | 188 |
| IL-8 | 5^′^-ATGAGTCTTAGAGGTCTGGGTG-3^′^ | 5^′^-ACAGTGAGGGCTAGGAGGG-3^′^ | 60.3°C | 114 |
| IL-10 | 5^′^-AATCCCTTTGATTTTGCC-3^′^ | 5^′^-GTGCCTTATCCTACAGTATGTG-3^′^ | 61.4°C | 256 |
| TGF-β | 5^′^-TTGGGACTTGTGCTCTAT-3^′^ | 5^′^-AGTTCTGCTGGGATGTTT-3^′^ | 55.9°C | 173 |
| NF-κB | 5^′^-GAAGAAGGATGTGGGAGATGC-3^′^ | 5^′^-TGTTGTCGTAGATGGGCTGAG-3^′^ | 62.3°C | 197 |
| IκB | 5^′^-TCTTGCCATTATTCACGAGG-3^′^ | 5^′^-TGTTACCACAGTCATCCACCA-3^′^ | 62.3°C | 197 |
| TOR | 5^′^-TCCCACTTTCCACCAACT-3^′^ | 5^′^-ACACCTCCACCTTCTCCA-3^′^ | 61.4°C | 177 |
| SOD1 | 5^′^-CGCACTTCAACCCTTACA-3^′^ | 5^′^-ACTTTCCTCATTGCCTCC-3^′^ | 61.5°C | 218 |
| GPx | 5^′^-GGGCTGGTTATTCTGGGC-3^′^ | 5^′^-AGGCGATGTCATTCCTGTTC-3^′^ | 61.5°C | 278 |
| Nrf2 | 5^′^-CTGGACGAGGAGACTGGA-3^′^ | 5^′^-ATCTGTGGTAGGTGGAAC-3^′^ | 62.5°C | 234 |
| Keap1 | 5^′^-TTCCACGCCCTCCTCAA-3^′^ | 5^′^-TGTACCCTCCCGCTATG-3^′^ | 63.0°C | 205 |
| β-Actin | 5^′^-GGCTGTGCTGTCCCTGTA-3^′^ | 5^′^-GGGCATAACCCTCGTAGAT-3^′^ | 61.4°C | 101 |
